# The genome sequence of an ichneumonid wasp, *Promethes sulcator* (Gravenhorst, 1829) (Hymenoptera: Ichneumonidae)

**DOI:** 10.12688/wellcomeopenres.26938.1

**Published:** 2026-07-04

**Authors:** Gavin R. Broad, Liam M. Crowley, Inez Januszczak, Chris Fletcher, Ian Barnes, Laura Sivess, Natural History Museum Genome Acquisition Lab, Darwin Tree of Life Barcoding Collective, Wellcome Sanger Institute Tree of Life Management, Samples and Laboratory team, Wellcome Sanger Institute Scientific Operations: Sequencing Operations, Wellcome Sanger Institute Tree of Life Core Informatics team, Tree of Life Core Informatics collective, Darwin Tree of Life Consortium

**Affiliations:** 1 Natural History Museum, London, England, UK; 2 University of Oxford, Oxford, England, UK

**Keywords:** Promethes sulcator, ichneumonid wasp, genome sequence, chromosomal, Hymenoptera

## Abstract

We present a genome assembly from an individual female *Promethes sulcator* (ichneumonid wasp; Arthropoda; Insecta; Hymenoptera; Ichneumonidae). The genome sequence has a total length of 278.74 megabases. Most of the assembly (99.67%) is scaffolded into 10 chromosomal pseudomolecules. The mitochondrial genome has also been assembled, with a length of 29.43 kilobases. This assembly was generated as part of the Darwin Tree of Life project, which produces genomes for eukaryotic species found in Britain and Ireland.

## Species taxonomy

Eukaryota; Opisthokonta; Metazoa; Eumetazoa; Bilateria; Protostomia; Ecdysozoa; Panarthropoda; Arthropoda; Mandibulata; Pancrustacea; Altocrustacea; Allotriocarida; Hexapoda; Insecta; Dicondylia; Pterygota; Neoptera; Eumetabola; Endopterygota; Hymenoptera; Apocrita; Ichneumonoidea; Ichneumonidae; Diplazontinae; *Promethes*; *Promethes sulcator* (Gravenhorst, 1829) (NCBI:txid662491).

## Background

*Promethes sulcator* is a small (~5 mm), widespread and common species of the ichneumonid subfamily Diplazontinae. As with other diplazontines, the mandible is tridentate, the upper tooth subdivided, and the first metasomal segment is parallel-sided. *Promethes* can be recognised by the smooth and shining face with paired vertical grooves in combination with the spiracle of the second metasomal tergite above the fold (below the fold in *Sussaba*). Among the five European *Promethes* ( Klopfstein, 2014), *P. sulcator* can be recognised by the slender antennae, partly reddish metasoma, black scutellum and lack of a subocular groove between the eye and mandible.

Found across much of the Palaearctic, India and the Nearctic ( Dasch, 1964; Diller, 1984; Klopfstein, 2014; Uchida, 1957), *P. sulcator* can be common and easily found in a variety of habitats. Specimens can be seen nectaring on flowers, are often swept from low vegetation and are regularly light-trapped, suggesting that this is a partly crepuscular or nocturnal species. Despite its wide range and abundance, little has been published on the biology of *P. sulcator.* As with most other Diplazontinae, *P. sulcator* is a parasitoid of aphid-feeding hoverflies (Diptera: Syrphidae), with host records from genera such as *Melanostoma*, *Platycheirus* and *Sphaerophoria* species (e.g. Scott, 1939; Maibach & Goeldlin de Tiefenau, 1991; Thirion, 1994), so apparently with a wide host range. All Diplazontinae are koinobiont endoparasitoids of Syrphidae larvae, emerging from the puparium. While some diplazontine genera oviposit in the eggs or very early instars of host hoverflies, others oviposit in older larvae, but it is unknown which stage of host *Promethes* oviposits in.

Males of *P. sulcator* have tyloids in the middle of the antenna which allow the antenna to coil around the female’s antenna during courtship ( Klopfstein *et al.*, 2010). *Promethes* and *Sussaba* form a rather distinctive, monophyletic group of Diplazontinae, with male antennae forming a single coil (rather than no coiling or two coils) in courtship and with a smooth, rather long face furnished with two grooves ( Klopfstein *et al.*, 2010, 2011). These two genera seem to be the sister group to the remaining Diplazontinae ( Klopfstein *et al.*, 2011). The genomes of *Sussaba pulchella* (Holmgren) and *Diplazon laetatorius* (Fabricius) were published recently ( Barnes *et al.*, 2025; Broad *et al.*, 2024), with more Diplazontinae in the Darwin Tree of Life pipeline.

## Methods

### Sample acquisition and DNA barcoding

**Figure 1.**
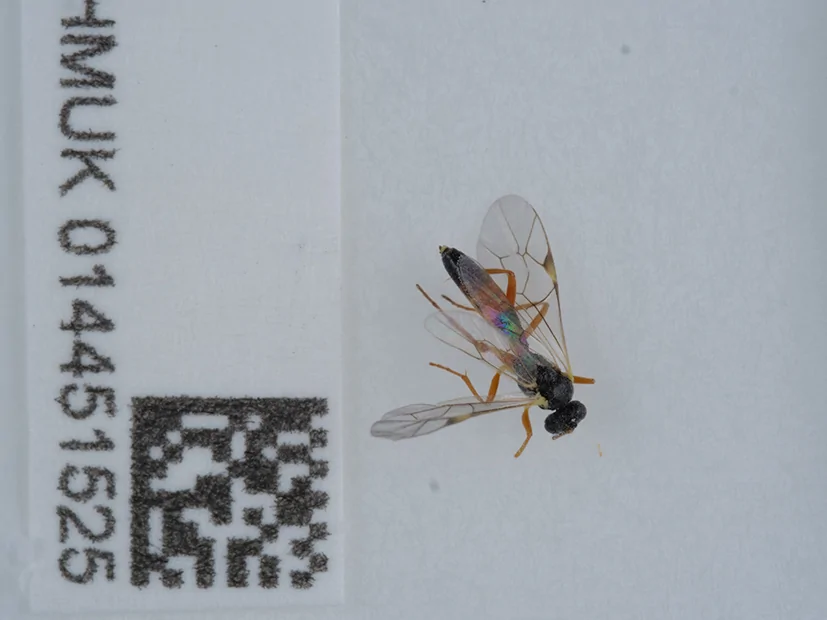
Photograph of the *Promethes sulcator* (iyProSulc1) specimen used for genome sequencing.

The specimen used for genome sequencing was an adult female *Promethes sulcator* (specimen ID NHMUK014451525, ToLID iyProSulc1; Figure 1), collected from Wytham Woods, Bert’s Pheasant Pen, England, UK (latitude 51.77, longitude −1.31) on 2021-09-02. A second specimen was used for Hi-C sequencing (specimen ID NHMUK014451615, ToLID iyProSulc2). It was collected on the same occasion as the iyProSulc1 specimen. The specimens were collected by Gavin Broad, Liam Crowley, Inez Januszczak, Chris Fletcher, and Ian Barnes and identified by Gavin Broad. Another specimen was used for RNA sequencing (specimen ID NHMUK010884542, ToLID iyProSulc3). It was collected from Bure Marshes National Nature Reserve, England, UK (latitude 52.69, longitude 1.46) on 2022-06-29. The specimen was collected by Laura Sivess and identified by Gavin Broad.

The initial identification was verified by an additional DNA barcoding process according to the framework developed by Twyford *et al.* (2024). A small sample was dissected from the specimen and stored in ethanol, while the remaining parts were shipped on dry ice to the Wellcome Sanger Institute (WSI) (see the protocol). The tissue was lysed, the COI marker region was amplified by PCR, and amplicons were sequenced and compared to the BOLD database, confirming the species identification ( Crowley *et al.*, 2023). Following whole genome sequence generation, the relevant DNA barcode region was also used alongside the initial barcoding data for sample tracking at the WSI ( Twyford *et al.*, 2024). The standard operating procedures for Darwin Tree of Life barcoding are available on protocols.io.

### Nucleic acid extraction

Detailed protocols for nucleic acid extraction developed at the Wellcome Sanger Institute (WSI) Tree of Life Core Laboratory are available on protocols.io ( Howard *et al.*, 2025). The iyProSulc1 specimen was weighed and triaged to determine the appropriate extraction protocol. For high molecular weight (HMW) DNA extraction, 2.0 mg of whole organism tissue was used. Tissue from the whole organism was homogenised by powermashing using a PowerMasher II tissue disruptor. HMW DNA was extracted in the WSI Scientific Operations core using the Automated MagAttract v2 protocol. We used centrifuge-mediated fragmentation to produce DNA fragments in the 8–10 kb range, following the Covaris g-TUBE protocol for ultra-low input (ULI). Sheared DNA was purified by manual SPRI (solid-phase reversible immobilisation).

The concentration of the sheared and purified DNA was assessed using a Nanodrop spectrophotometer and Qubit Fluorometer using the Qubit dsDNA High Sensitivity Assay kit. Fragment size distribution was evaluated by running the sample on the FemtoPulse system. For this sample, the final post-shearing DNA had a Qubit concentration of 1.3 ng/μL and a yield of 75.40 ng, with a fragment size of 13.8 kb.

RNA was extracted from whole organism tissue of the iyProSulc3 specimen in the Tree of Life Laboratory at the WSI using the RNA Extraction: Automated MagMax™ *mir*Vana protocol. The RNA concentration was assessed using a Nanodrop spectrophotometer and a Qubit Fluorometer using the Qubit RNA Broad-Range Assay kit. Analysis of the integrity of the RNA was done using the Agilent RNA 6000 Pico Kit and Eukaryotic Total RNA assay.

### PacBio HiFi library preparation and sequencing

Library preparation and sequencing were performed at the WSI Scientific Operations core. Prior to library preparation, the DNA was fragmented to ~10 kb. Ultra-low-input (ULI) libraries were prepared using the PacBio SMRTbell® Express Template Prep Kit 2.0 and gDNA Sample Amplification Kit. Samples were normalised to 20 ng DNA. Single-strand overhang removal, DNA damage repair, and end-repair/A-tailing were performed according to the manufacturer’s instructions, followed by adapter ligation. A 0.85× pre-PCR clean-up was carried out with Promega ProNex beads.

The DNA was evenly divided into two aliquots for dual PCR (reactions A and B), both following the manufacturer’s protocol. A 0.85× post-PCR clean-up was performed with ProNex beads. DNA concentration was measured using a Qubit Fluorometer v4.0 (Thermo Fisher Scientific) with the Qubit HS Assay Kit, and fragment size was assessed on an Agilent Femto Pulse Automated Pulsed Field CE Instrument (Agilent Technologies) using the gDNA 55 kb BAC analysis kit. PCR reactions A and B were then pooled, ensuring a total mass of ≥500 ng in 47.4 μl.

The pooled sample underwent another round of DNA damage repair, end-repair/A-tailing, and hairpin adapter ligation. A 1× clean-up was performed with ProNex beads, followed by DNA quantification using the Qubit and fragment size analysis using the Agilent Femto Pulse. Size selection was performed on the Sage Sciences PippinHT system, with target fragment size determined by Femto Pulse analysis (typically 4–9 kb). Size-selected libraries were cleaned with 1.0× ProNex beads and normalised to 2 nM before sequencing.

The sample was sequenced on a Revio instrument (Pacific Biosciences, California, USA). Prepared libraries selected for multiplexing were pooled based on genome size, library molarity, and the intended plex level. Primers were annealed and polymerases were bound to generate circularised complexes, following the manufacturer’s instructions. Complexes were purified using SMRTbell beads, diluted to the Revio loading concentration, and spiked with a Revio sequencing internal control. The pooled libraries were sequenced on a Revio 25 M Plex SMRT cell in a 4-plex run. SMRT Link software (Pacific Biosciences), a web-based workflow manager, was used to configure and monitor the run and to carry out primary and secondary data analysis.

### Hi-C

***Sample preparation and crosslinking***

The Hi-C sample was prepared from 20–50 mg of frozen whole organism tissue from the iyProSulc2 sample using the Arima-HiC v2 kit (Arima Genomics). Following the manufacturer’s instructions, tissue was fixed and DNA crosslinked using TC buffer to a final formaldehyde concentration of 2%. The tissue was homogenised using the Diagnocine Power Masher-II. Crosslinked DNA was digested with a restriction enzyme master mix, biotinylated, and ligated. Clean-up was performed with SPRISelect beads before library preparation. DNA concentration was measured with the Qubit Fluorometer (Thermo Fisher Scientific) and Qubit HS Assay Kit. The biotinylation percentage was estimated using the Arima-HiC v2 QC beads.

***Hi-C library preparation and sequencing***

Biotinylated DNA constructs were fragmented using a Covaris E220 sonicator and size selected to 400–600 bp using SPRISelect beads. DNA was enriched with Arima-HiC v2 kit Enrichment beads. End repair, A-tailing, and adapter ligation were carried out with the NEBNext Ultra II DNA Library Prep Kit (New England Biolabs), following a modified protocol where library preparation occurs while DNA remains bound to the Enrichment beads. Library amplification was performed using KAPA HiFi HotStart mix and a custom Unique Dual Index (UDI) barcode set (Integrated DNA Technologies). Depending on sample concentration and biotinylation percentage determined at the crosslinking stage, libraries were amplified with 10–16 PCR cycles. Post-PCR clean-up was performed with SPRISelect beads. Libraries were quantified using the AccuClear Ultra High Sensitivity dsDNA Standards Assay Kit (Biotium) and a FLUOstar Omega plate reader (BMG Labtech).

Prior to sequencing, libraries were normalised to 10 ng/μL. Normalised libraries were quantified again to create equimolar and/or weighted 2.8 nM pools. Pool concentrations were checked using the Agilent 4200 TapeStation (Agilent) with High Sensitivity D500 reagents before sequencing. Sequencing was performed using paired-end 150 bp reads on the Illumina NovaSeq X.

### RNA library preparation and sequencing

Libraries were prepared using the NEBNext® Ultra II Directional RNA Library Prep Kit for Illumina (New England Biolabs), following the manufacturer’s instructions. Poly(A) mRNA was isolated from 100 ng of total RNA using oligo (dT) beads, converted to cDNA, and uniquely indexed; 14 PCR cycles were performed. Libraries were prepared with an intended fragment size of 100–300 bp. Libraries were quantified, normalised, and pooled equimolarly to a final concentration of 2.8 nM before dilution to 150 pM for loading. Sequencing was carried out on the Illumina NovaSeq X, generating paired-end reads.

### Genome assembly

Prior to assembly of the PacBio HiFi reads, a database of *k*-mer counts ( *k* = 31) was generated from the filtered reads using FastK. GenomeScope2 ( Ranallo-Benavidez *et al.*, 2020) was used to analyse the *k*-mer frequency distributions, providing estimates of genome size, heterozygosity, and repeat content.

The HiFi reads were assembled using Hifiasm ( Cheng *et al.*, 2021) with the --primary option. Haplotypic duplications were identified and removed using purge_dups ( Guan *et al.*, 2020). The Hi-C reads ( Rao *et al.*, 2014) were mapped to the primary contigs using bwa-mem2 ( Vasimuddin *et al.*, 2019), and the contigs were scaffolded in YaHS ( Zhou *et al.*, 2023) with the --break option for handling potential misassemblies. The scaffolded assemblies were evaluated using Gfastats ( Formenti *et al.*, 2022), BUSCO ( Manni *et al.*, 2021) and MerquryFK ( Rhie *et al.*, 2020).

The mitochondrial genome was assembled using MitoHiFi ( Uliano-Silva *et al.*, 2023).

### Assembly curation

The assembly was decontaminated using the Assembly Screen for Cobionts and Contaminants ( ASCC) pipeline. TreeVal was used to generate the flat files and maps for use in curation. Manual curation was conducted primarily in PretextView and HiGlass ( Kerpedjiev *et al.*, 2018). Scaffolds were visually inspected and corrected as described by Howe *et al*. (2021).

Manual corrections included 149 breaks and 354 joins. This reduced the scaffold count by 39.4% and increased the scaffold N50 by 18.8%. The curation process is described at https://gitlab.com/wtsi-grit/rapid-curation . PretextSnapshot was used to generate a Hi-C contact map of the final assembly.

### Assembly quality assessment

The MerquryFK tool ( Rhie *et al.*, 2020) was run in a Singularity container ( Kurtzer *et al.*, 2017) to evaluate *k*-mer completeness and assembly quality for the primary and alternate haplotypes using the *k*-mer database ( *k* = 31) computed prior to genome assembly. The analysis outputs included assembly QV scores and completeness statistics.

The genome was analysed using the BlobToolKit pipeline, a Nextflow implementation of the earlier Snakemake version ( Challis *et al.*, 2020). The pipeline aligns PacBio reads using minimap2 ( Li, 2018) and SAMtools ( Danecek *et al.*, 2021) to generate coverage tracks. It runs BUSCO ( Manni *et al.*, 2021) using lineages identified from the NCBI Taxonomy ( Schoch *et al.*, 2020). For the three domain-level lineages, BUSCO genes are aligned to the UniProt Reference Proteomes database ( Bateman *et al.*, 2023) using DIAMOND blastp ( Buchfink *et al.*, 2021). The genome is divided into chunks based on the density of BUSCO genes from the closest taxonomic lineage, and each chunk is aligned to the UniProt Reference Proteomes database with DIAMOND blastx. Sequences without hits are chunked using seqtk and aligned to the NT database with blastn ( Altschul *et al.*, 1990). The BlobToolKit suite consolidates all outputs into a blobdir for visualisation. The BlobToolKit pipeline was developed using nf-core tooling ( Ewels *et al.*, 2020) and MultiQC ( Ewels *et al.*, 2016), with containerisation through Docker ( Merkel, 2014) and Singularity ( Kurtzer *et al.*, 2017).

## Genome sequence report

### Sequence data

**Figure 2.**
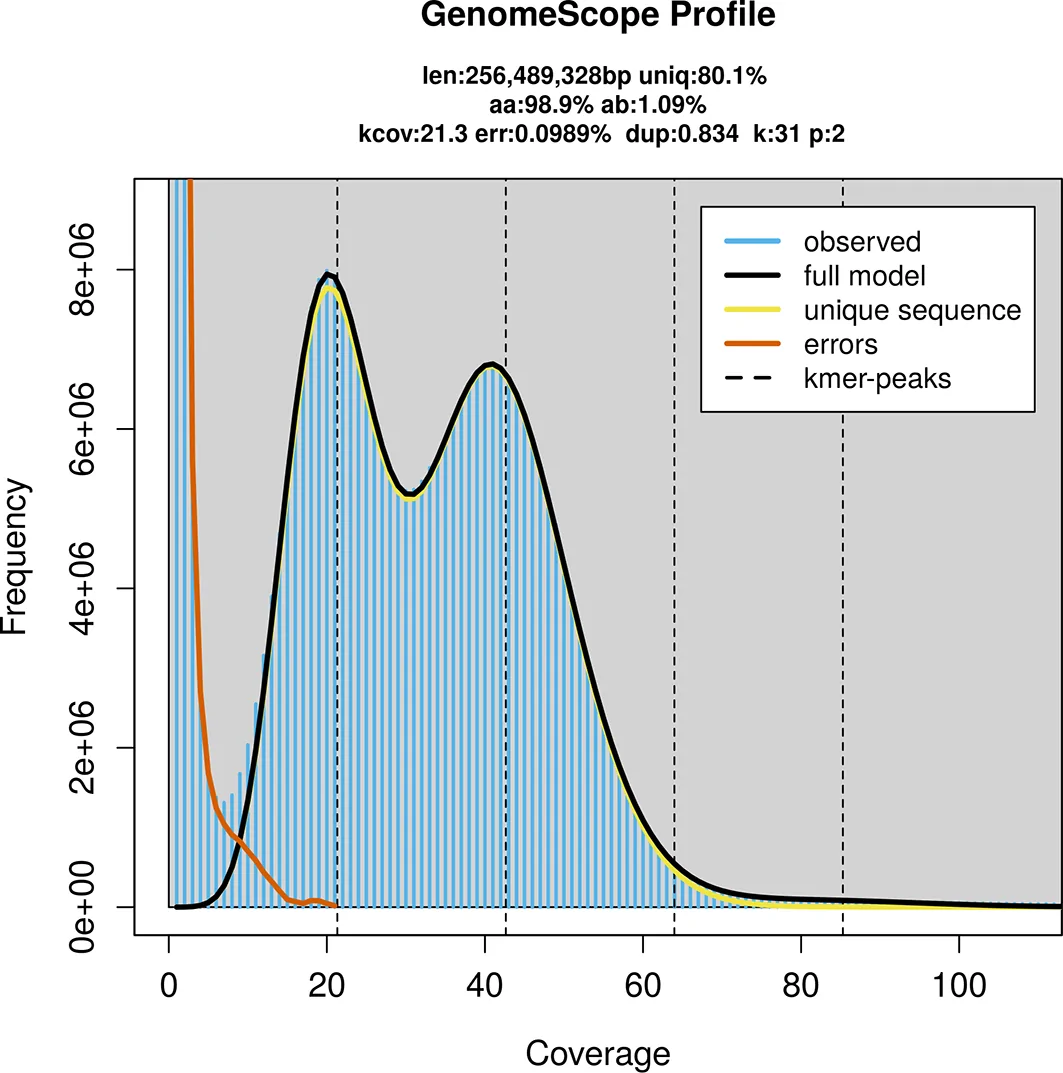
Frequency distribution of *k*-mers generated using GenomeScope2. The plot shows observed and modelled *k*-mer spectra, providing estimates of genome size, heterozygosity, and repeat content based on unassembled sequencing reads.

PacBio sequencing of the *Promethes sulcator* specimen generated 11.58 Gb (gigabases) from 0.93 million reads, which were used to assemble the genome. GenomeScope2.0 analysis estimated the haploid genome size at 256.49 Mb, with a heterozygosity of 1.09% and repeat content of 20.03% ( Figure 2). These estimates guided expectations for the assembly. Based on the estimated genome size, the sequencing data provided approximately 43× coverage. Hi-C sequencing produced 81.39 Gb from 269.50 million reads, which were used to scaffold the assembly. RNA sequencing data were also generated and are available in public sequence repositories. Table 1 summarises the specimen and sequencing details.

**Table 1.** Specimen and sequencing data for *Promethes sulcator* (BioProject PRJEB84440).

Platform	PacBio HiFi	Hi-C	RNA-seq
**ToLID**	iyProSulc1	iyProSulc2	iyProSulc3
**Specimen ID**	NHMUK014451525	NHMUK014451615	NHMUK010884542
**BioSample (source individual)**	SAMEA110021798	SAMEA111458164	SAMEA114805923
**BioSample (tissue)**	SAMEA14448535	SAMEA111458236	SAMEA114806097
**Tissue**	whole organism	whole organism	whole organism
**Instrument**	Revio	Illumina NovaSeq X	Illumina NovaSeq X
**Run accessions**	ERR14151160	ERR14172012	ERR14379161
**Read count total**	0.93 million reads	269.50 million read pairs	39.63 million read pairs
**Base count total**	11.58 Gb	81.39 Gb	11.97 Gb

### Assembly statistics

The primary haplotype was assembled, and contigs corresponding to an alternate haplotype were also deposited in INSDC databases. The final assembly has a total length of 278.74 Mb in 59 scaffolds, with 460 gaps, and a scaffold N50 of 38.96 Mb ( Table 2).

**Table 2.** Genome assembly data for *Promethes sulcator.*

Genome assembly	Primary assembly
**Assembly name**	iyProSulc1.1
**Assembly accession**	GCA_965283325.1
**Alternate haplotype accession**	GCA_965283305.1
**Assembly level**	chromosome
**Span (Mb)**	278.74
**Number of chromosomes**	10
**Number of contigs**	519
**Contig N50**	1.27 Mb
**Number of scaffolds**	59
**Scaffold N50**	38.96 Mb
**Organelles**	Mitochondrial genome: 29.43 kb

**Figure 3.**
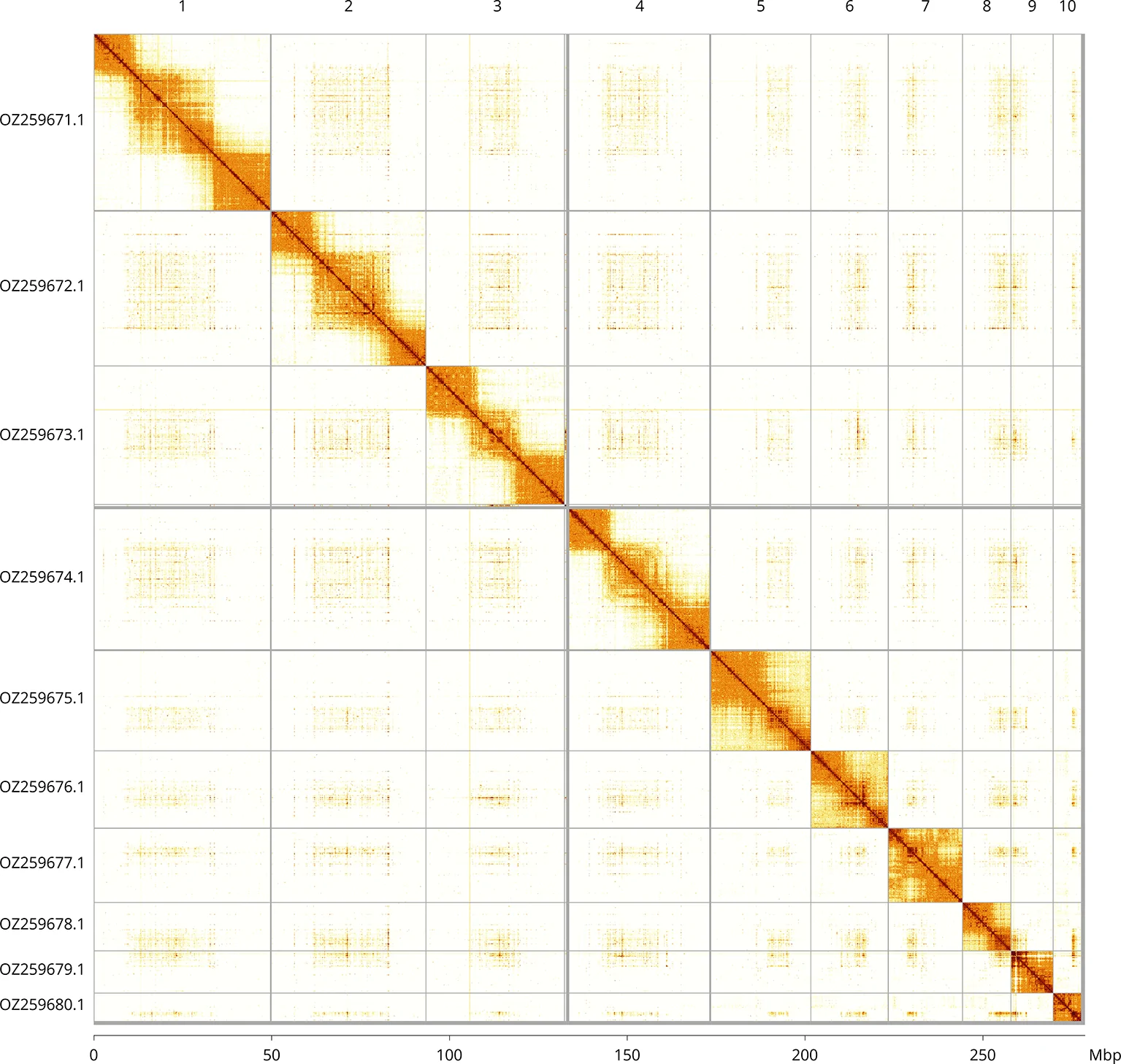
Hi-C contact map of the *Promethes sulcator* genome assembly. Assembled chromosomes are shown in order of size and labelled along the axes, with a megabase scale shown below. The plot was generated using PretextSnapshot.

Most of the assembly sequence (99.67%) was assigned to 10 chromosomal-level scaffolds. These chromosome-level scaffolds, confirmed by Hi-C data, are named according to size ( Figure 3; Table 3). The order and orientation of scaffolds are uncertain on chromosome 3 from ~14.73–25.34 Mb, Chromosome 4 from ~11.58–17.21 Mb and Chromosome 8 from ~9.76–13.62 Mb.

**Table 3.** Chromosomal pseudomolecules in the primary genome assembly of *Promethes sulcator* iyProSulc1.

INSDC accession	Molecule	Length (Mb)	GC%
OZ259671.1	1	49.91	41.50
OZ259672.1	2	43.50	41.50
OZ259673.1	3	40.19	42
OZ259674.1	4	39.88	42.50
OZ259675.1	5	28.16	41.50
OZ259676.1	6	21.83	41.50
OZ259677.1	7	20.85	41.50
OZ259678.1	8	13.64	42
OZ259679.1	9	11.81	42
OZ259680.1	10	8.03	42

The mitochondrial genome was also assembled (length 29.43 kb, OZ259681.1). This sequence is included as a contig in the multifasta file of the genome submission and as a standalone record.

### Assembly quality metrics

**Figure 4.**
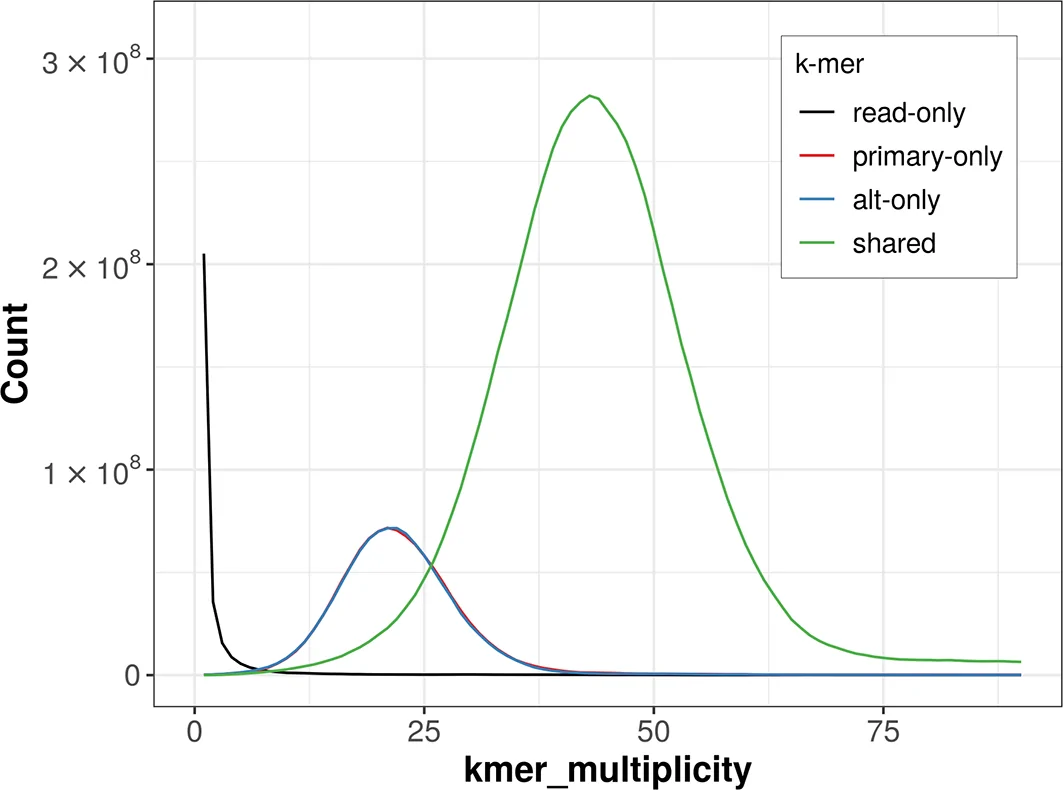
Evaluation of *k*-mer completeness using MerquryFK. This plot illustrates the recovery of *k*-mers from the original read data in the final assemblies. The horizontal axis represents *k*-mer multiplicity, and the vertical axis shows the number of *k*-mers. The black curve represents *k*-mers that appear in the reads but are not assembled. The green curve corresponds to *k*-mers shared by both haplotypes, and the red and blue curves show *k*-mers found only in one of the haplotypes.

The combined primary and alternate assemblies achieve an estimated QV of 58.4. The *k*-mer completeness is 81.25% for the primary assembly, 81.15% for the alternate haplotype, and 99.56% for the combined assemblies ( Figure 4).

**Figure 5.**
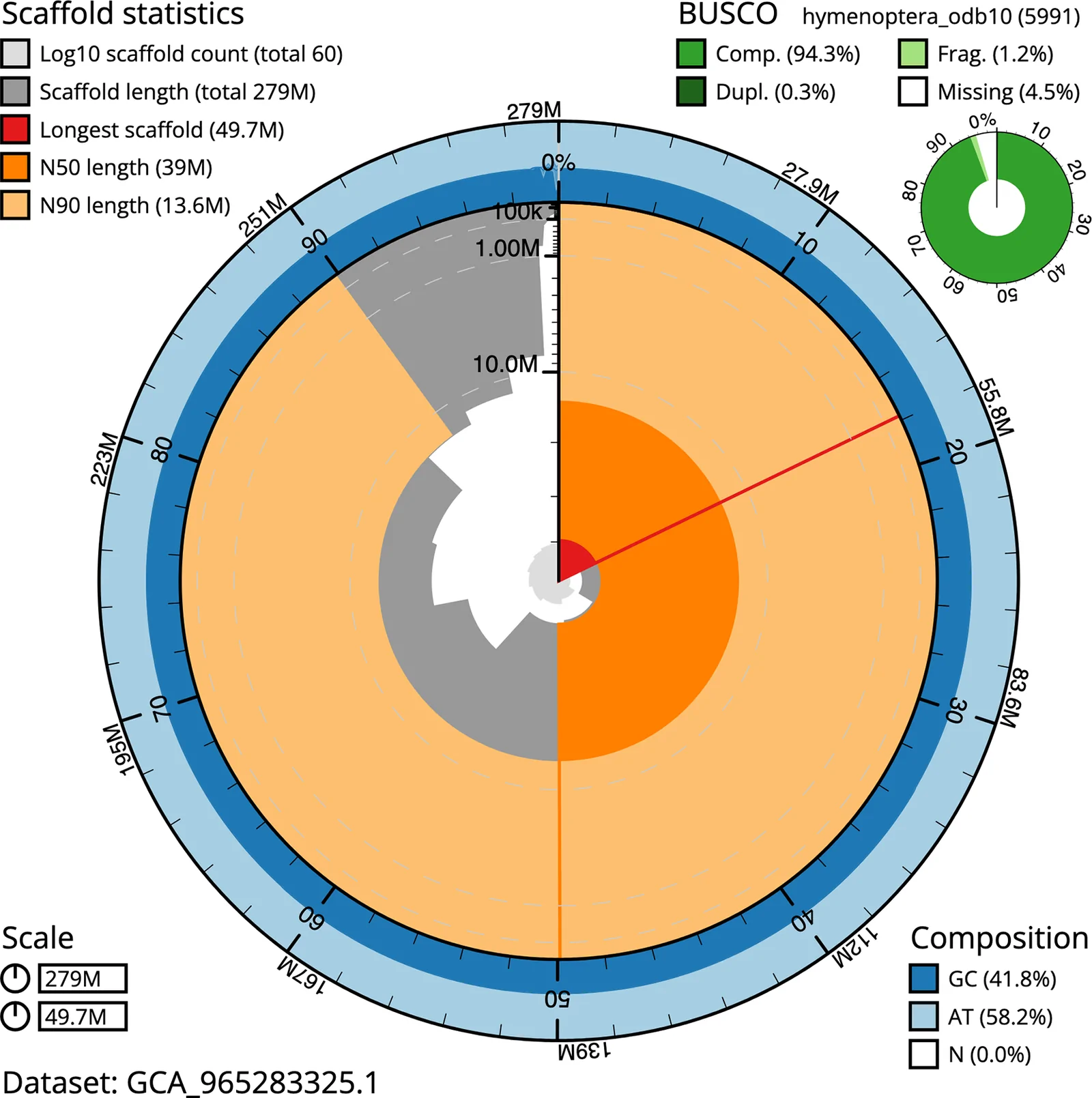
Assembly metrics for iyProSulc1.1. The BlobToolKit snail plot provides an overview of assembly metrics and BUSCO gene completeness. The circumference represents the length of the whole genome sequence, and the main plot is divided into 1 000 bins around the circumference. The outermost blue tracks display the distribution of GC, AT, and N percentages across the bins. Scaffolds are arranged clockwise from longest to shortest and are depicted in dark grey. The longest scaffold is indicated by the red arc, and the deeper orange and pale orange arcs represent the N50 and N90 lengths. A light grey spiral at the centre shows the cumulative scaffold count on a logarithmic scale. A summary of complete, fragmented, duplicated, and missing BUSCO genes in the hymenoptera_odb10 set is presented at the top right. An interactive version of this figure can be accessed on the BlobToolKit viewer.

**Figure 6.**
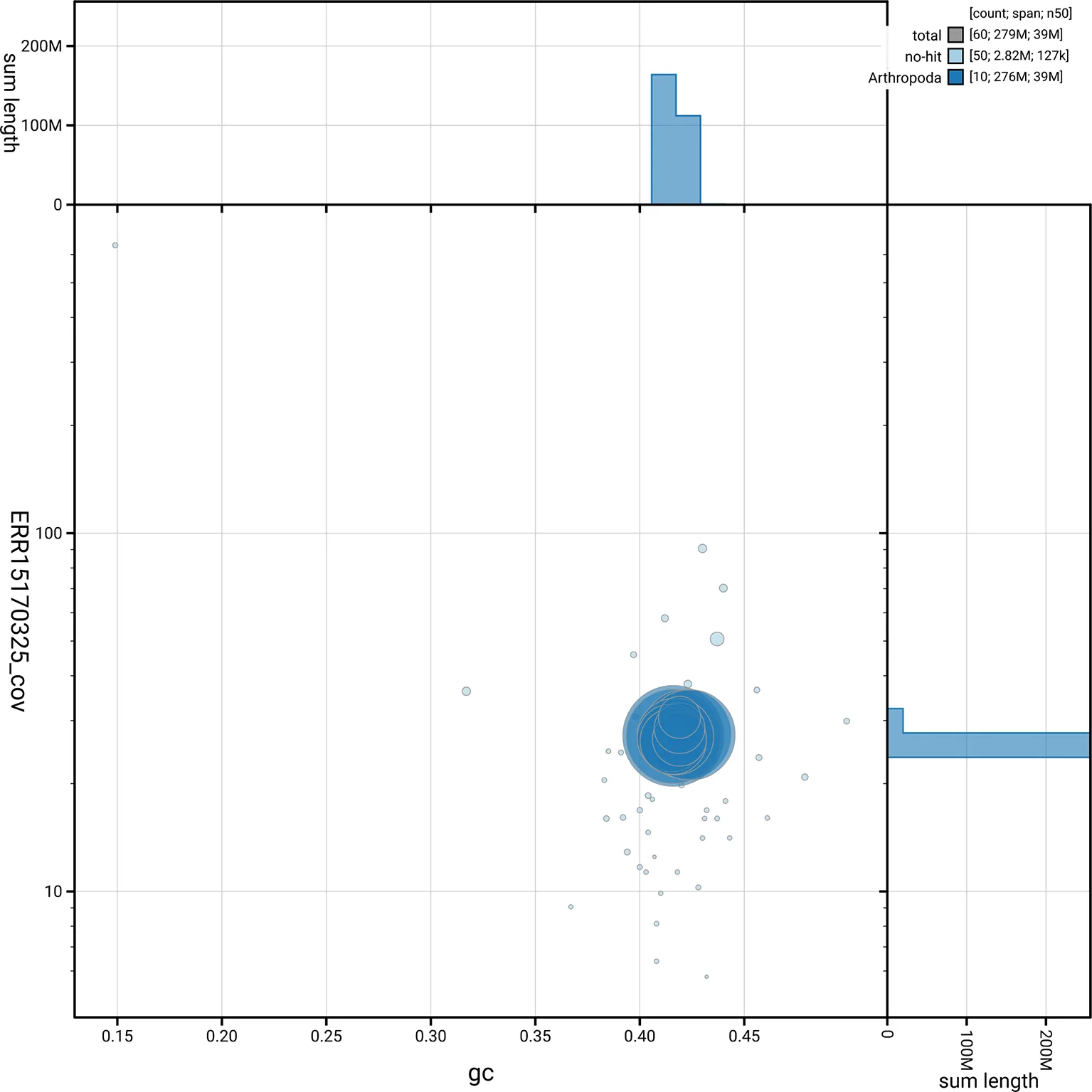
BlobToolKit blob plot for iyProSulc1.1. The plot shows base coverage (vertical axis) and GC content (horizontal axis). The circles represent scaffolds, with the size proportional to scaffold length and the colour representing phylum membership. The histograms along the axes display the total length of sequences distributed across different levels of coverage and GC content. An interactive version of this figure is available on the BlobToolKit viewer.

BUSCO v.5.7.1 analysis using the hymenoptera_odb10 reference set ( *n* = 5 991) identified 94.3% of the expected gene set (single = 94.0%, duplicated = 0.3%). The snail plot in Figure 5 summarises the scaffold length distribution and other assembly statistics for the primary assembly. The blob plot in Figure 6 shows the distribution of scaffolds by GC proportion and coverage.

Table 4 lists the assembly metric benchmarks adapted from Rhie *et al.* (2021) and the Earth BioGenome Project Report on Assembly Standards. The EBP metric, calculated for the primary assembly, is **6.C.Q58**, meeting the recommended 6.C.Q40 reference standard.

**Table 4.** Earth Biogenome Project summary metrics for the *Promethes sulcator* assembly.

Measure	Value	Benchmark
EBP summary (primary)	6.C.Q58	6.C.Q40
Contig N50 length	1.27 Mb	≥ 1 Mb
Scaffold N50 length	38.96 Mb	= chromosome N50
Consensus quality (QV)	Primary: 58.1; alternate: 58.6; combined: 58.4	≥ 40
*k*-mer completeness	Primary: 81.25%; alternate: 81.15%; combined: 99.56%	≥ 95%
BUSCO	C:94.3% [S:94.0%, D:0.3%], F:1.2%, M:4.5%, n:5 991	S > 90%; D < 5%
Percentage of assembly assigned to chromosomes	99.67%	≥ 90%

**Notes:** The EBP summary uses log10(Contig N50); chromosome-level (C) or log10(Scaffold N50); Q (Merqury QV). BUSCO: C = complete; S = single-copy; D = duplicated; F = fragmented; M = missing; n = orthologues

**Table 5.** Software versions and sources used for *Promethes sulcator.*

Software	Version	Source
BLAST	2.14.0	ftp://ftp.ncbi.nlm.nih.gov/blast/executables/blast+/
BlobToolKit	4.4.5	https://github.com/blobtoolkit/blobtoolkit
BUSCO	5.7.1	https://gitlab.com/ezlab/busco
bwa-mem2	2.2.1	https://github.com/bwa-mem2/bwa-mem2
DIAMOND	2.1.8	https://github.com/bbuchfink/diamond
fasta_windows	0.2.4	https://github.com/tolkit/fasta_windows
FastK	1.1	https://github.com/thegenemyers/FASTK
GenomeScope2.0	2.0.1	https://github.com/tbenavi1/genomescope2.0
Gfastats	1.3.6	https://github.com/vgl-hub/gfastats
Hifiasm	0.19.8-r603	https://github.com/chhylp123/hifiasm
HiGlass	1.13.4	https://github.com/higlass/higlass
MerquryFK	1.1.0-c1	https://github.com/thegenemyers/MERQURY.FK
Minimap2	2.28-r1209	https://github.com/lh3/minimap2
MitoHiFi	3	https://github.com/marcelauliano/MitoHiFi
MultiQC	1.14; 1.17 and 1.18	https://github.com/MultiQC/MultiQC
Nextflow	24.10.4	https://github.com/nextflow-io/nextflow
PretextSnapshot	0.0.5	https://github.com/sanger-tol/PretextSnapshot
PretextView	1.0.3	https://github.com/sanger-tol/PretextView
samtools	1.21	https://github.com/samtools/samtools
sanger-tol/ascc	0.1.0	https://github.com/sanger-tol/ascc
sanger-tol/blobtoolkit	v0.7.1	https://github.com/sanger-tol/blobtoolkit
sanger-tol/curationpretext	1.4.2	https://github.com/sanger-tol/curationpretext
Seqtk	1.3	https://github.com/lh3/seqtk
Singularity	3.9.0	https://github.com/sylabs/singularity
TreeVal	1.4.0	https://github.com/sanger-tol/treeval
YaHS	1.2.2	https://github.com/c-zhou/yahs

## Author information

Contributors are listed at the following links:

• Members of the Natural History Museum Genome Acquisition Lab
• Members of the Darwin Tree of Life Barcoding collective
• Members of the Wellcome Sanger Institute Tree of Life Management, Samples and Laboratory team
• Members of Wellcome Sanger Institute Scientific Operations – Sequencing Operations
• Members of the Wellcome Sanger Institute Tree of Life Core Informatics team
• Members of the Tree of Life Core Informatics collective
• Members of the Darwin Tree of Life Consortium

## Wellcome Sanger Institute – Legal and Governance

The materials that have contributed to this genome note have been supplied by a Darwin Tree of Life Partner. The submission of materials by a Darwin Tree of Life Partner is subject to the **‘Darwin Tree of Life Project Sampling Code of Practice’**, which can be found in full on the Darwin Tree of Life website. By agreeing with and signing up to the Sampling Code of Practice, the Darwin Tree of Life Partner agrees they will meet the legal and ethical requirements and standards set out within this document in respect of all samples acquired for, and supplied to, the Darwin Tree of Life Project. Further, the Wellcome Sanger Institute employs a process whereby due diligence is carried out proportionate to the nature of the materials themselves, and the circumstances under which they have been/are to be collected and provided for use. The purpose of this is to address and mitigate any potential legal and/or ethical implications of receipt and use of the materials as part of the research project, and to ensure that in doing so we align with best practice wherever possible. The overarching areas of consideration are:

• Ethical review of provenance and sourcing of the material
• Legality of collection, transfer and use (national and international)

Each transfer of samples is further undertaken according to a Research Collaboration Agreement or Material Transfer Agreement entered into by the Darwin Tree of Life Partner, Genome Research Limited (operating as the Wellcome Sanger Institute), and in some circumstances, other Darwin Tree of Life collaborators.

## Data Availability

European Nucleotide Archive: Promethes sulcator. Accession number PRJEB84440; https://identifiers.org/ena.embl/PRJEB84440. The genome sequence is released openly for reuse. The *Promethes sulcator* genome sequencing initiative is part of the Darwin Tree of Life Project (PRJEB40665) and the Sanger Institute Tree of Life Programme (PRJEB43745). All raw sequence data and the assembly have been deposited in INSDC databases. The genome will be annotated using available RNA-Seq data and presented through the Ensembl pipeline at the European Bioinformatics Institute. Raw data and assembly accession identifiers are reported in Tables 1 and 2. Production code used in genome assembly at the WSI Tree of Life is available at https://github.com/sanger-tol . Table 5 lists software versions used in this study.
