# Supplementary material for: The genome sequence of an ichneumonid wasp, Promethes sulcator (Gravenhorst, 1829) (Hymenoptera: Ichneumonidae)
Source: Wellcome Open Res. 2026 Jul 4;11:412. [Version 1] doi: 10.12688/wellcomeopenres.26938.1 (PMC13560794; doi:10.12688/wellcomeopenres.26938.1)
Supplement: Peer review history

# Reviewer report: Reviewer response for version 1

**DOI:** 10.21956/wellcomeopenres.29657.r162484

**Published:** 2026-08-05

**Authors:** Andrew D. (Andy) Austin

**Affiliations:** 1 University of Adelaide, Adelaide, Australia

There is little I need to say about this paper. It follows the same format as previous wasp genome papers by the same authors. Although I am not expert in the details of the sequencing methods, they are clearly presented in detail and and well explained.  This paper will add to a growing body of information that will be important for understanding the evolution of parasitic wasps and functional aspects of their genomes

Are sufficient details of methods and materials provided to allow replication by others?

Yes

Is the rationale for creating the dataset(s) clearly described?

Yes

Are the datasets clearly presented in a useable and accessible format?

Yes

Are the protocols appropriate and is the work technically sound?

Yes

Reviewer Expertise:

Wasps phylogenetics and evolution

I confirm that I have read this submission and believe that I have an appropriate level of expertise to confirm that it is of an acceptable scientific standard.

# Reviewer report: Reviewer response for version 1

**DOI:** 10.21956/wellcomeopenres.29657.r160833

**Published:** 2026-07-24

**Authors:** Christopher Douglas Robert Wyatt

**Affiliations:** 1 University College London, London, UK

Background is concise and informative, fitting to the wasps's ecological importance. This will be a very useful genome for the community.

 Maybe you could add better , more functional keywords like "parasitoid". Didn't see keywords in other genome notes.

 Methods are consistent with other genome note reports. HiC contact map shows distinct pseudo-molecule chromosomes. So seems complete.

Are sufficient details of methods and materials provided to allow replication by others?

Yes

Is the rationale for creating the dataset(s) clearly described?

Yes

Are the datasets clearly presented in a useable and accessible format?

Yes

Are the protocols appropriate and is the work technically sound?

Yes

Reviewer Expertise:

genomics, transcriptomics, biofinformatics

I confirm that I have read this submission and believe that I have an appropriate level of expertise to confirm that it is of an acceptable scientific standard.
